# Flavonoid metabolism is involved in regulating the growth of winter wheat upon rehydration

**DOI:** 10.3389/fpls.2025.1693576

**Published:** 2026-02-02

**Authors:** Xuejing Liu, Baozhong Yin, Chong Shang, Xiaoyuan Bao, Li Wang, Tao Wang, Wenchao Zhen

**Affiliations:** 1 College of Mining Engineering, Hebei Industrial Technology Institute of Mine Ecological Remediation, North China University of Science and Technology, Tangshan, China; 2 College of Agronomy, Hebei Agricultural University, Baoding, China; 3 Key Laboratory of North China Water-saving Agriculture, Ministry of Agriculture and Rural Affairs, Baoding, China; 4 State Key Laboratory of North China Crop Improvement and Regulation, Baoding, China; 5 State Key Laboratory of Aridland Crop Science, Gansu Agricultural University, Lanzhou, China

**Keywords:** flavonoid metabolism, rehydration, transcriptomic, winter wheat, yield composition

## Abstract

**Introduction:**

Extensive research has been conducted on water- limited irrigation strategies and yield component variations in winter wheat (Triticum aestivum L.). However, limited understanding exists regarding the nuanced responses of winter wheat canopies and gene expressions to rehydration events.

**Methods:**

A field investigation was carried out throughout the winter wheat growing season from 2018 to 2020. Four distinct irrigation schedules were implemented, with water supplementation carefully synchronized with irrigation timing to match the appearance of the third, fourth, fifth, and sixth leaves. Further investigation into the molecular mechanisms of winter wheat rehydration using RNA- seq and ultra- performance liquid chromatography- mass spectrometry (UPLC- MS).

**Results and discussion:**

Our findings show that delayed rehydration results in reduced total water use across all treatment groups during the reproductive growth period, especially from jointing to flowering. A consistent pattern of reduction was observed in leaf area index (LAI), biomass at maturity (BAM), and plant height as rehydration was progressively delayed. The analysis found no statistically significant differences in phenotypic traits among winter wheat at the four- leaf stage before irrigation. In contrast, delaying rehydration until the fifth- leaf stage in spring had a noticeable impact on phenotypic traits. Implementing delayed rehydration at the four- leaf stage increased grain yield by 8. 31% to 51.23. 23%, mainly through three key yield components: more spikes, optimized grains per spike, and higher 1000- grain weight. Interestingly, the increase in 1000- grain weight was inversely related to total grain quantity after postponed rehydration. Transcriptomic and metabolomic analyses showed that postponed rehydration was associated with flavonoid biosynthesis pathways. Notably, the gene related to dihydrokaempferol- known to be involved in phenylpropanoid, flavonol, and flavone biosynthesis- showed a significant positive correlation with naringenin, chrysin, taxifolin, and prunin. Chlorogenic acid and luteolin also exhibited strong positive correlations with various agronomic traits, such as kernel number and 1000- grain weight. These results suggest the presence of a potential molecular regulator at a critical developmental stage, offering new insights into the mechanisms influencing crop yield under water- restricted irrigation conditions.

## 1 Introduction

As a globally vital cereal crop, winter wheat (Triticum aestivum L.) faces persistent production constraints due to water scarcity (Zeng et al., 2023). Climate change-induced environmental fluctuations, particularly altered precipitation patterns and increased drought stress throughout crop development stages, may exacerbate yield reduction in wheat cultivation (Cao et al., 2024; Wan et al., 2022). Strategies to enhance plant drought tolerance and adapt to future climates are urgently required to sustain higher cereal yields. Limited irrigation has gained recognition as an effective strategy for enhancing crop yields while mitigating water scarcity (Tan et al., 2023). One key methodology is determining the optimal irrigation schedule for critical stages of wheat growth. Link water deficit to yield losses across different crop growth stages, including establishment, vegetative growth, flowering, and yield formation, as well as their consecutive combinations. The flowering stage and its combinations are particularly sensitive to water stress compared to other phases (Monteleone et al., 2023). Nevertheless, existing irrigation recommendations demonstrate considerable spatial variability throughout winter wheat cultivation zones, primarily attributable to geographical differences in water availability. The physiological and biochemical pathways through which regulated deficit irrigation affects crop growth and metabolic processes remain incompletely characterized (Yang et al., 2022; Wang et al., 2023). Consequently, understanding the variability of water deficits during different growth stages of winter wheat in various regions of the Chinese winter wheat belt helps develop regionally appropriate water-saving practices. Elucidating the balance between crop growth and stress responses in mitigating drought stress across varying soil water regimes is crucial for providing valuable empirical insights to enhance and sustain wheat productivity under adverse conditions.

Crop responses to drought stress exhibit significant dependence on three key meteorological parameters: event duration, intensity, and frequency of drought events, as supported by extensive research. During periods of water limitation, crops optimize the balance between vegetative growth and yield components (Ali et al., 2018). Substantial research has documented the yield compensation phenomenon resulting from regulated deficit irrigation during fertility stages across major cereal crops, including wheat, maize, and soybeans (Sintaha et al., 2022; He et al., 2023; Liu et al., 2024; Wu P.Q et al., 2024). Ongoing scientific discourse persists regarding ideal water restriction timing, primarily due to regional variations in edaphic and climatic conditions. For winter wheat, the sensitive period for deficit irrigation has been reported to be from jointing to anthesis (Feng et al., 2023; Li et al., 2023). Research by Liu et al. (2024) demonstrated the critical role of water management during wheat vegetative growth stages in mitigating the adverse effects of subsequent drought conditions following flowering. Investigations on deficit irrigation at anthesis showed that supplemental irrigation during this stage significantly increased yield (Fang et al., 2021; Li et al., 2023). The soil moisture content was significantly influenced by the various treatments applied at different growth stages of wheat. Winter wheat subjected to drought priming exhibited a stress imprint that enhanced its response to subsequent water deficit conditions during later growth stages. This improvement was evidenced by the progression of test weight, grain yield, plant-level water use efficiency, irrigation water use efficiency, and relative yield (Katohar et al., 2023). Morphological plasticity in wheat exhibits pronounced sensitivity to water limitation, manifesting through reduction in leaf size and plant height, which represent specific adaptations to drought stress. These structural modifications contribute to optimized population architecture and improved canopy configuration, thereby enhancing light interception and utilization (Liu et al., 2024). According to He et al. (2023), water deficit exerts detrimental effects on crop yield through dual mechanisms of growth inhibition and impaired photoassimilate translocation. However, increased efficiency in transporting organic matter to the grains helps sustain high yield levels (Ali et al., 2018).

Improving the distribution of assimilation to the young ear is a key strategy for preventing kernel loss, increasing the number of grains, and boosting overall yield under drought stress. Nevertheless, the associated genes and metabolic pathways remain poorly characterized (H. T. Li et al., 2023). Elucidating the molecular mechanisms behind the responses and compensatory effects of regulated deficit irrigation constitutes a crucial research priority. Flavonoid compounds function as inhibitors of auxin translocation in plants, thereby modulating shoot elongation. These secondary metabolites potentially promote carbohydrate remobilization from the stem to the ear, thereby improving floret survival and ultimately contributing to enhanced grain number and crop yield (Franco et al., 2015; N. Q. Dong and Lin, 2021). Flavonoids played an important role in wheat, which exhibits varying levels of drought tolerance under drought stress and during rehydration. The increased biosynthetic capability of amino acids and the ability to scavenge reactive oxygen species (ROS) result from enhanced antioxidant activities and elevated flavonoid levels, which may underlie the characteristics of drought tolerance (Lv et al., 2022). Metabolome and transcriptome analyses revealed that drought-tolerant wheat varieties exhibit elevated levels of flavonoid and phenolic acid metabolites and genes in response to drought stress. Thus, the metabolism of these compounds appears to be pivotal for the drought resistance of wheat seedlings (X. R. Guo et al., 2025). Abscisic acid, trehalose 6-phosphate, and flavonoids inhibit stem elongation and the movement of assimilates into the kernel. These compounds participate comprehensively in drought tolerance processes throughout the pre-anthesis phase in Zea mays, establishing a systemic regulatory mechanism for drought tolerance (Gao et al., 2023).

While physiological and morphological research alone cannot systematically elucidate the complex mechanisms of drought response, multi-omics joint analysis offers an effective approach to comprehensively reveal the mechanisms underlying plant responses to drought stress. However, to the best of our knowledge, there is a paucity of studies examining the drought stress response mechanisms in wheat, particularly in relation to flavonoids and their role in drought tolerance. The current understanding of the interplay between flavonoids and developmental processes in drought-stressed wheat is incomplete, especially regarding the underlying physiological regulation. Further research is necessary to ascertain whether this response contributes to increased yield. To address this gap, a two-year field experiment was conducted, incorporating four distinct phases of water-limited irrigation, to investigate the metabolic mechanisms linking nutrient allocation and yield formation that enhance drought tolerance in winter wheat. A comprehensive analysis of organ-specific phenotypic and metabolic responses to drought provides critical insights for optimizing winter wheat yield under conditions of water-limited irrigation.

## 2 Materials and methods

### 2.1 Plant material and rehydration treatment

The experiments were conducted at the Shenzhou Experimental Station of the Institute of Dry Farming (geographic coordinates: 37°54′N, 115°42′E; Shenzhou City, Hebei Province, China) from 2018 to 2020 during the winter wheat seasons. The study utilized Hengguan 35 (HG35), a winter wheat genotype with contrasting drought tolerance and representing one of the predominant genotypes cultivated across the North China Plain. Field experiments were conducted at a research station with warm temperate continental monsoon climate conditions, with a mean annual temperature of 13.4°C and an average annual precipitation of 480.7 mm. Soil analysis revealed a medium loam classification with measured bulk density averaging 1.56 g/cm³ across experimental plots. Volumetric water content at field capacity within a 0–200 cm soil profile reached 39.35%. Soil parameters, including the content of organic matter, available nitrogen, and the amounts of available phosphorus and potassium at the 0–20 cm depth, were quantified as 16.7 g/kg, 95.28 mg/kg, 31.85 mg/kg, and 159.51 mg/kg, respectively. Winter wheat was planted on 13 October in both 2018 and 2019. Each plot measured 4 m × 10 m, and the seeds were planted at a density of 3.75 × 10^6^/ha. Prior to planting, a total of 120 kg/ha of nitrogen, 120 kg/ha of potassium, and 105 kg/ha of phosphorus fertilizers were applied. An additional 120 kg/ha of nitrogen was applied with the initial spring irrigation. Other cultivation practices were based on high-yield field methods.

The field experiment implemented four irrigation periods, with three replications per treatment, to ensure statistical reliability. The present study employed a randomized block design. These were represented by L3, L4, L5, and L6, respectively. Soil moisture measurements were conducted at 20-cm depth increments throughout the 0–200 cm profile, utilizing a Trimer Pico 64 portable time-domain reflectometry (TDR) soil moisture meter (IMIKO, Germany). Irrigation was provided to each plot until the soil relative moisture content of the 0–60 cm layer reached 80% of the field capacity volumetric water content.

### 2.2 Water consumption

The evapotranspiration of winter wheat was calculated following established methodology (Bandyopadhyay et al., 2005; Kuang et al., 2020):

(1) ET=P+I+W–R–D+ΔSWC

The equation employed the following parameters: ET for evapotranspiration (mm), P for precipitation (mm) throughout the winter wheat growing stage, and I for irrigation (mm). Groundwater recharge (W, mm) was excluded from analysis because the water table depth exceeded 5 m. ΔSWC quantified soil water consumption (mm) for the winter wheat growing season within the 0–200 cm soil profile. Surface runoff (R, mm) and percolation (D, mm) were omitted from calculations due to the absence of substantial rainfall events throughout the experimental period.

### 2.3 Quantifications of agronomic traits

Leaf morphological measurements were conducted on twenty randomly selected wheat plants per plot at 10-day intervals beginning at the jointing stage, utilizing a standard ruler to measure leaf length and maximum leaf width. The LAI was defined by the following equation, as proposed by Li et al.,(2022):

(2) $$\text{Leaf area }({\text{cm}}^{2})=\text{leaf length }(\text{cm})\times \text{leaf width }(\text{cm})\times 0.83$$

(3) $$\text{Leaf area index }(\text{LAI})=\text{green leaf area }({\text{m}}^{2})\text{ per stem}\times \text{tillers }({\text{m}}^{-2})$$

Samples of aboveground plants from each plot of double rows measuring 1 m were collected at the jointing stage, anthesis stage, and maturity stage. Subsequently, the samples were subjected to a 48-hour drying process at 80°C in an oven until constant weight was stabilized.

### 2.4 Grain yields and its components

Growth analysis involved sampling representative 1.5 m × 1.0 m areas in each plot to quantify three yield components: spike length (SL), the number of sterile spikelets, and the total number of spikelets per hectare. The calculation of the 1000-grain weight was derived from the yield sample. Grain yield (GY, kg/ha) was calculated by weighing the grain harvested with a plot harvester and adjusting for a moisture content of 12.5%.

### 2.5 RNA-Seq and bioinformatics analysis

Leaf samples for RNA sequencing were collected from wheat plants eight days after irrigation under L3, L4, L5, and L6 conditions (X. Liu et al., 2022). Control leaf samples originated from corresponding non-irrigated treatments, sampled simultaneously with each irrigation treatment, and represented by L3CK, L4CK, L5CK, and L6CK. One biological replicate was created by combining ten independent plants, and three biological replicates were sampled at each time point. Following collection, the samples were frozen in liquid nitrogen and stored at -80°C.

RNA sequencing was performed by Novogene Co. (Beijing, China) using a NEBNext^®^ Ultra™ RNA Library Prep Kit for Illumina^®^ (New England Biolabs, USA). The library preparations were sequenced on an Illumina Novaseq platform, and the quality of the library was evaluated using an Agilent Bioanalyzer 2100 system. Subsequent bioinformatics processing included calculation of Q20 values, Q30 values, and GC content from filtered sequencing data. Alignment of processed reads against the International Wheat Genome Sequencing Consortium reference genome was performed with HISAT2 software. The adjusted p-value was set to < 0.05, and the |log2FoldChange| was set to ≥ 1 for determining DEGs, which were then subjected to k-means clustering. Data visualization incorporated heatmap generation and principal component analysis (PCA) through R packages. Functional annotation of DEGs involved Kyoto Encyclopedia of Genes and Genomes (KEGG) pathway and Gene Ontology (GO) enrichment analyses implemented in R using hypergeometric tests. The raw transcriptome data were deposited at NCBI BioProject PRJNA1216897.

### 2.6 Metabolomic analysis

Ultra-high-performance liquid chromatography (UHPLC) with tandem mass spectrometry (MS/MS) analyses was conducted using a Vanquish UHPLC system in conjunction with an Orbitrap Q Exactive™ HF mass spectrometer, both from Thermo Fisher Scientific, at Novogene Co., Ltd. (Beijing, China). UHPLC-MS/MS analyses were conducted using a Vanquish UHPLC system and an Orbitrap Q Exactive™ HF mass spectrometer at Novogene Co., Ltd. Samples were injected onto a Hypersil Gold column with a 17-minute linear gradient at 0.2 mL/min. In positive mode, eluents were 0.1% formic acid in water and methanol; in negative mode, they were 5 mM ammonium acetate (pH 9.0) and methanol. The solvent gradient was: 2% B for 1.5 min, 2-100% B over 12 min, 100% B for 14 min, 100-2% B at 14.1 min, and 2% B at 17 min. The mass spectrometer operated in positive/negative mode with a spray voltage of 3.2 kV, capillary temperature of 320 °C, sheath gas flow of 40 arb, and aux gas flow of 10 arb.

Statistical analysis employed Student’s t-test to calculate statistical significance (P-value). Differential metabolites were identified based on three criteria: VIP value exceeding 1, P < 0.05, and fold change ≥ 2 or ≤ 0.5. Metabolite annotation utilized three reference databases: KEGG (https://www.genome.jp/kegg/pathway.html), HMDB (https://hmdb.ca/metabolites), and LIPIDMaps (http://www.lipidmaps.org/). Multivariate analysis incorporated both partial least squares discriminant analysis (PLS-DA) and PCA, implemented through the metaX metabolomics data processing software.

### 2.7 Data processing and statistical analysis

Statistical evaluation employed SPSS Statistics 22.0 (IBM, New York, NY, USA) with results expressed as mean ± standard error (SE). One-way ANOVA was used, followed by t-tests applying a 5% significance level. Graphs were created using Origin version 2022 (OriginLab, Northampton, MA) combined with FigDraw software for graphical representation.

## 3 Results

### 3.1 Water consumption characteristics in response to postponed rehydration

**Figure 1:**
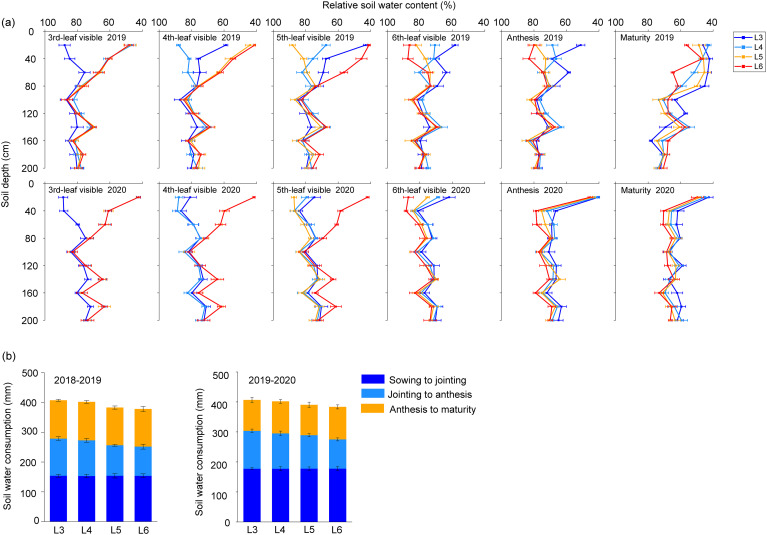
Relative soil water content **(a)** and water consumption **(b)** upon postponed rehydration. Values are mean ± standard error.

The relative soil water content at depths of 0–60 cm (RSWC60) exhibited the most pronounced variation between irrigation treatments, whereas the soil water content at depths of 60–200 cm demonstrated a relatively weaker variation (**Figure 1a**). During spring growth stages, the non-irrigated control exhibited 57% RSWC60 before the fourth-leaf irrigation, while RSWC60 at five- and six-leaf stages showed values below 50%, indicating moderate drought stress. Contrastingly, the RSWC60 was relatively high during the flowering and maturity stages for L5 and L6. Third-leaf irrigation produced inverse moisture dynamics, whereas fourth-leaf irrigation treatment soils remained suboptimal until maturity, with delayed attainment of the 60% moisture threshold reflecting minimal drought stress.

Delayed rehydration decreased overall water consumption across all experimental groups during the reproductive phase (**Figure 1b**; Equation 1). During the developmental phase spanning jointing to flowering, all treatments with delayed rehydration exhibited reduced water use, with the L3 and L4 treatment groups demonstrating less pronounced decreases. These treatments (L3 and L4) maintained significantly elevated water consumption levels, showing an average increment of 19.40 mm compared to the L5 and L6 treatments. Notably, water use patterns showed no significant differences between treatments from flowering through maturity.

### 3.2 Agronomic traits response to postponed rehydration

**Figure 2:**
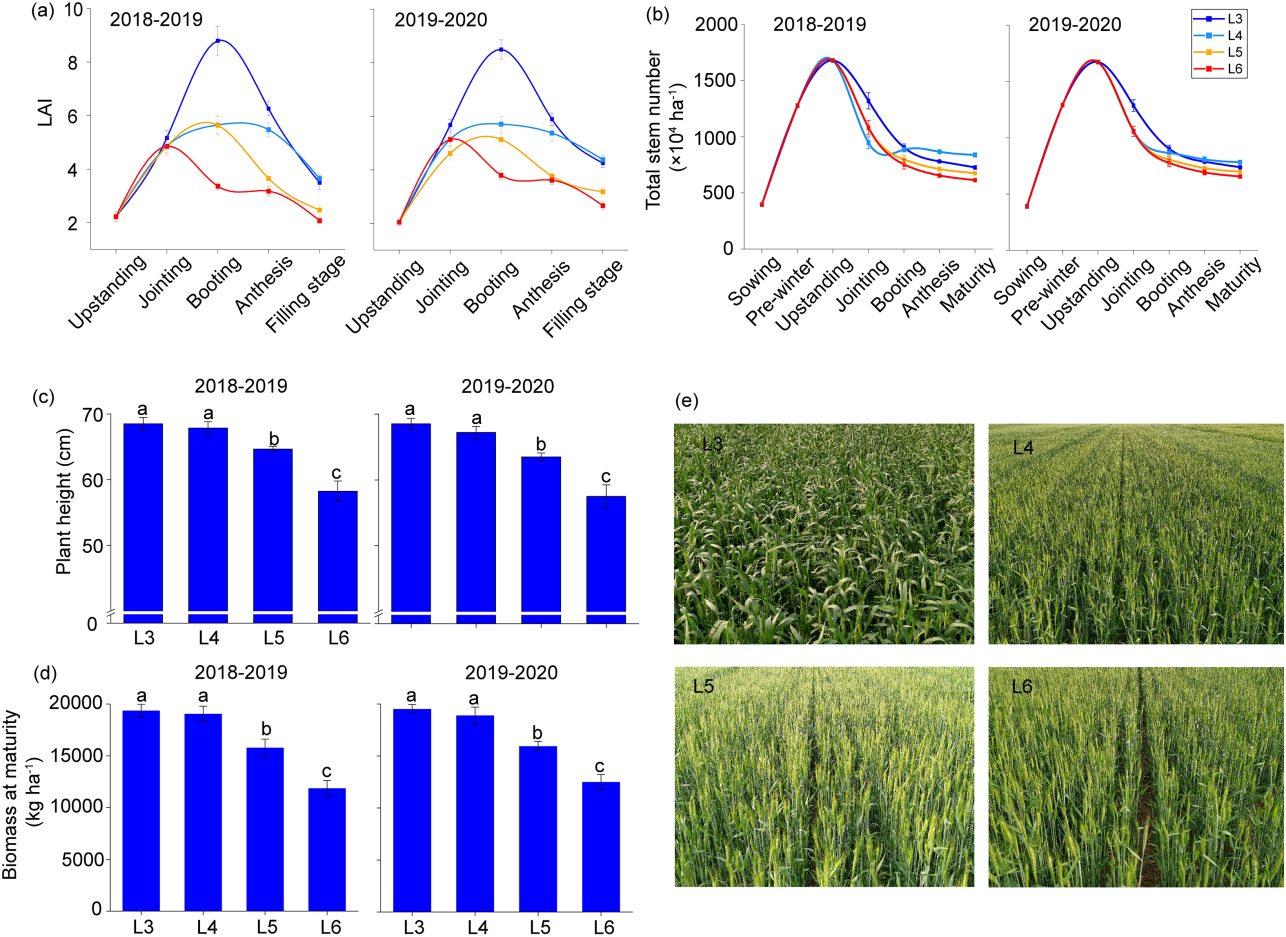
LAI **(a)**, total stem number **(b)**, plant height **(c)**, and biomass at maturity **(d)** upon postponed rehydration. Values are means ± standard errors. Figure **(e)** depicts the field landscape of winter wheat at the booting stage.

**Figure 2a** illustrates the dynamics of LAI across four irrigation regimes from standing through filling stages (Equation 2, 3). All delayed rehydration treatments reached maximum LAI values during booting, with L3 treatment exhibiting the highest measurement. Compared to L3, LAI decreased by 34.3%, 37.8%, and 58.5% at the booting stage for L4, L5, and L6, respectively. Post-flowering analysis revealed converging LAI values among treatments, except for a pronounced decrease in L5.

The L3 treatment demonstrated a maximal stem number (SN) during the initial growth phase of winter wheat (before the jointing stage) (**Figure 2b**). This pattern shifted at the booting stage, with L4 treatment maintaining superior stem retention through maturity due to a lower rate of tiller mortality. Postponed rehydration progressively diminished both plant height and BAM (**Figures 2c–e**), though L3 and L4 showed comparable values. Similar to LAI, plant height and BAM decreased in response to delayed rehydration, especially in the L5 treatment. These results suggest that delaying rehydration until the fifth-leaf stage in spring significantly affects the phenotypic characteristics of winter wheat.

### 3.3 Spike development and yield components in response to postponed rehydration

**Figure 3:**
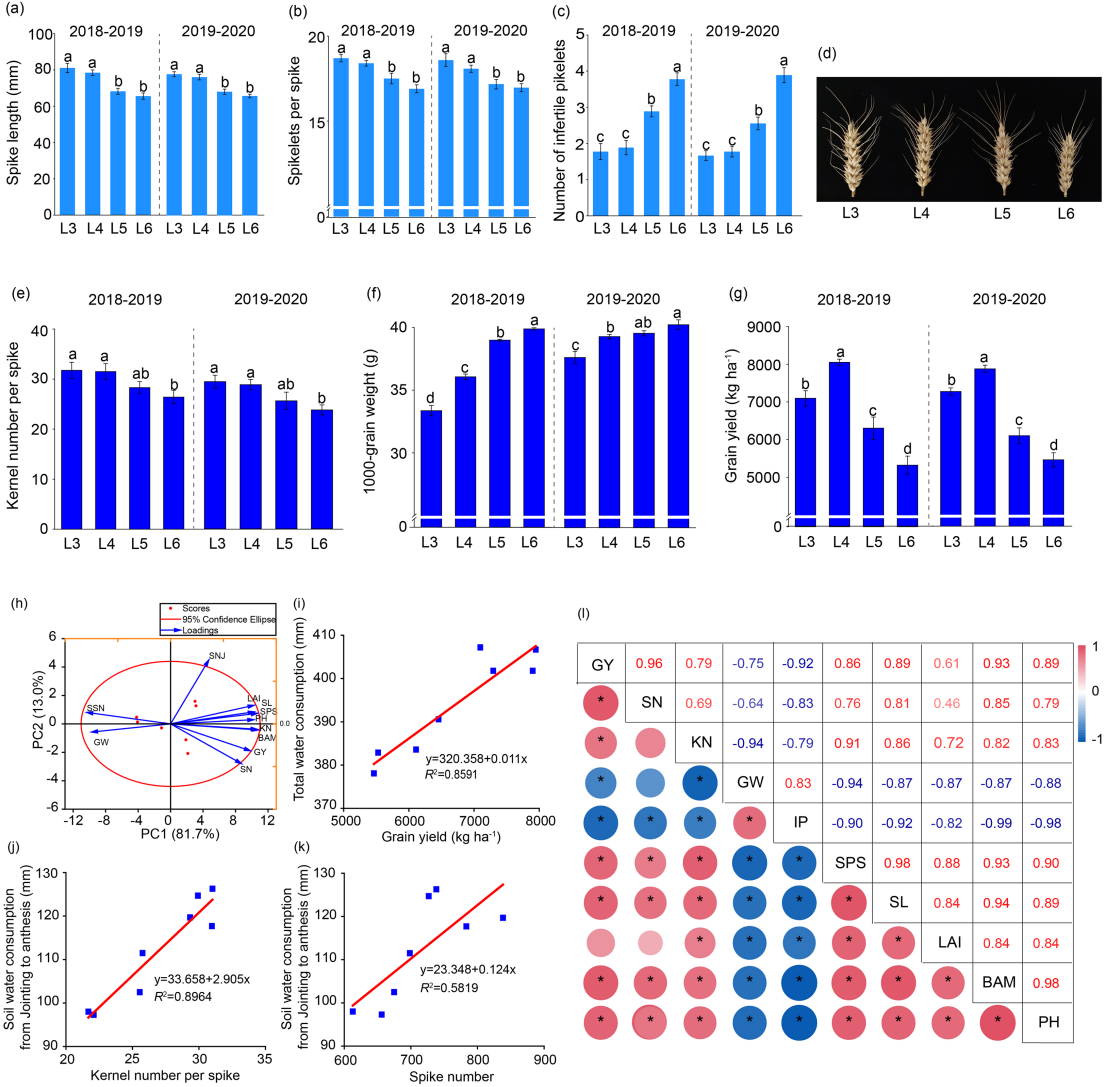
Characteristics of ear development and yield formation of winter wheat **(a–g)**, and biplot **(h)** and correlation analysis **(i–l)** on different traits of postponed rehydration. GY, SN, KN, GW, IP, SPS, SL, LAI, BAM, PH, SNJ and SSN represent grain yield, spike number, number of kernels per spike, 1000-grain weight, number of sterile spikelets, spikelets per spike, spike length, leaf area index, biomass at maturity, plant height, number of stems at jointing, number of sterile spikelets, respectively. * Indicates significant differences between the means of the different treatment groups at P < 0.05.

SL and the number of spikelets per spike (SPS) declined notably in L5 and L6 compared to L3 and L4, while no significant difference occurred between L3 and L4 (**Figures 3a, b**). The relatively higher number of infertile spikelets (IS) in the L5 and L6 treatments increased by 1.8-2.0 spikelets and correlated with a 3.45 to 5.35 reduction in grains per spike (**Figures 3c, d**). Postponed rehydration resulted in a significant improvement in 1000-grain weight, increasing by 1.20 g at L4, 2.04 g at L5, and 4.77 g at L6. Meanwhile, kernel number decreased by 3.20-5.35 on average over two years compared to L3 (**Figures 3e, f**). Compared to L3/L5/L6, the L4 treatment demonstrated a grain yield enhancement ranging from 8.31% to 51.23% (**Figure 3g**). This yield improvement was predominantly associated with two key factors: the maximum SN (**Figure 2b**) and a greater balance of GN and GW in L4.

PCA was performed to characterize ten winter wheat traits under postponed rehydration. The resulting biplot displayed characteristic distribution of factor 1 and factor 2 across scatterplot quadrants (**Figure 3h**). Parallel or proximal vector alignment signified strong positive trait correlations, whereas opposing vector directions indicated pronounced negative correlations. A weak correlation was indicated by vectors that were laterally oriented. The first principal component accounted for 81.7% of the total contribution rate across four irrigation stages, while PC1 was primarily represented by spike number, BAM, and kernel number per spike. Winter wheat yield exhibited significant positive correlations with multiple parameters: total water consumption, the number of grains per spike, the number of spikes, and soil moisture content (SWC) between the jointing and anthesis stages (**Figures 3i–k**). Further analysis revealed strong positive associations with grain yield and its components: spike number, 1000-grain weight, and plant height (**Figure 3l**). In general, the indirect effects of delayed rehydration tended to be attributed to an increase in the harvest index, effective spike number, and BAM. Among yield-limiting factors, sterile spikelet count emerged as the predominant negative determinant.

### 3.4 Transcriptome profiles in response to postponed rehydration

**Figure 4:**
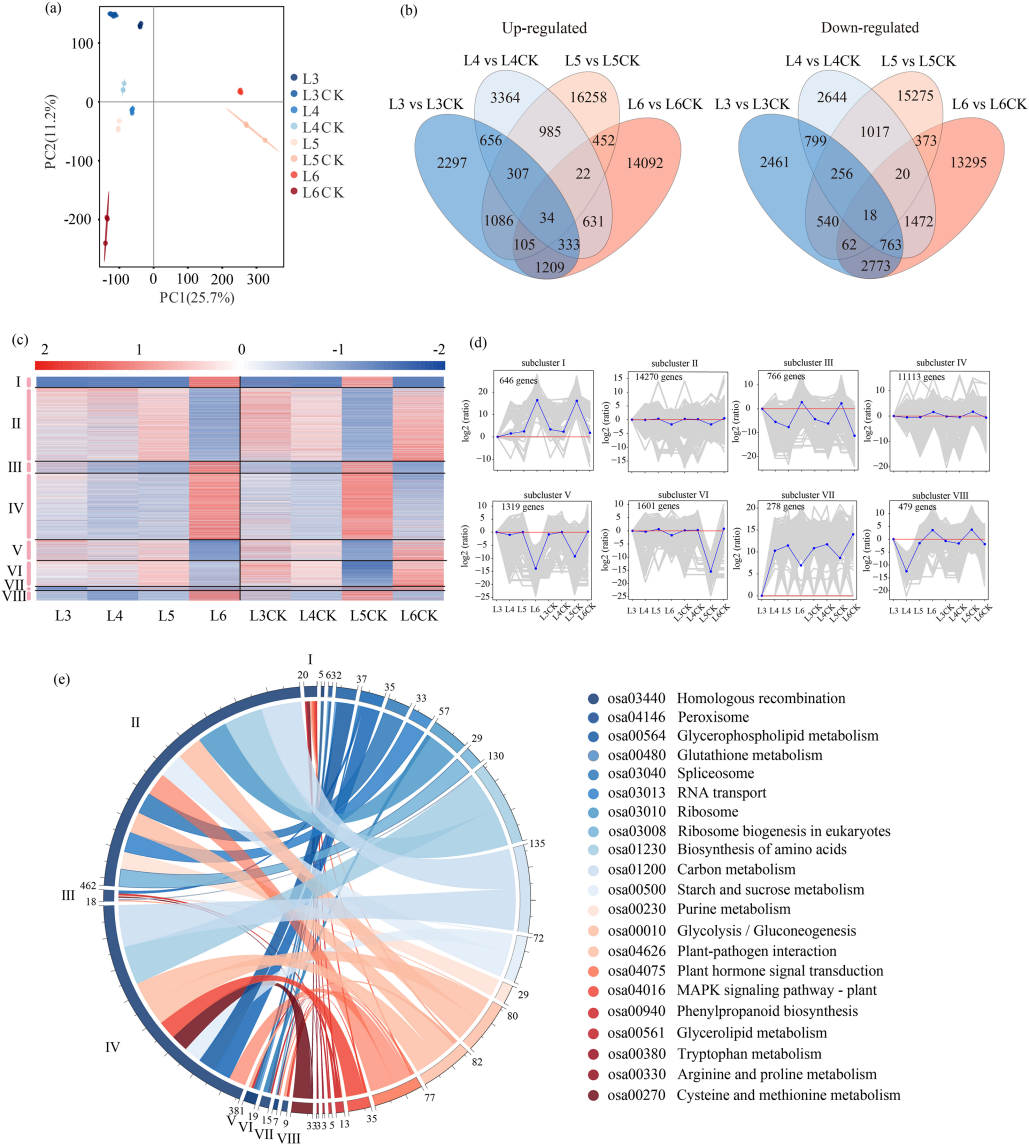
Transcriptome analysis of winter wheat in response to rehydration and prolonged drought stress. **(a)** Principal component analysis of total gene expressions in all the sequenced RNA libraries were performed to assess the correlations between the different samples. **(b)** Venn diagram illustrating the number of rehydration-drought shared, rehydration-specific, and drought-specific DEGs. K-means clustering **(c)** and expression level trends **(d)** of DEGs. **(e)** KEGG annotations of shared and specific DEGs in rehydration and drought.

Genome sequence mapping successfully identified 83,559 genes. PCA of transcriptome data showed tight clustering of biological replicates by rehydration stage, demonstrating high reproducibility in overall gene expression patterns (**Figure 4a**). Differential expression analysis employing DESeq2 software with stringent thresholds (adjusted p-value < 0.05 and |log2FoldChange| >1) revealed significant DEGs. Compared to the control group, the L3, L4, L5, and L6 treatments exhibited 2297, 3364, 16258, and 14092 specific upregulated genes, respectively, accompanied by 2461, 2644, 15275, and 13295 downregulated genes (**Figure 4b**). A K-means cluster analysis was conducted on differentially expressed genes identified in at least two comparison groups (**Figure 4c**), which segregated the transcriptome into eight distinct expression profiles. Clusters I and VII demonstrated progressive upregulation correlating with prolonged rehydration delay, whereas Clusters III and VIII presented an expression trend of first decreasing and then increasing (**Figure 4d**).

KEGG pathway annotation was conducted for eight gene clusters identified through transcriptomic analysis (**Figure 4e**). KEGG functional enrichment analysis indicated that the DEGs were primarily enriched in three key metabolic pathways: starch and sucrose metabolism, amino acid biosynthesis, and carbon metabolism. Cluster-specific analysis demonstrated distinct functional specialization, with Clusters I and VIII showing significant enrichment in phytohormone signaling and peroxisome, while Clusters II and III mainly participated in starch and sucrose metabolism and amino acid biosynthesis. Additional pathway analysis detected notable enrichment of phenylpropanoid biosynthesis pathways across five gene clusters.

### 3.5 Transcriptomic and metabolomic analysis reveals flavonoid metabolism-related pathways adapting to postponed rehydration

**Figure 5:**
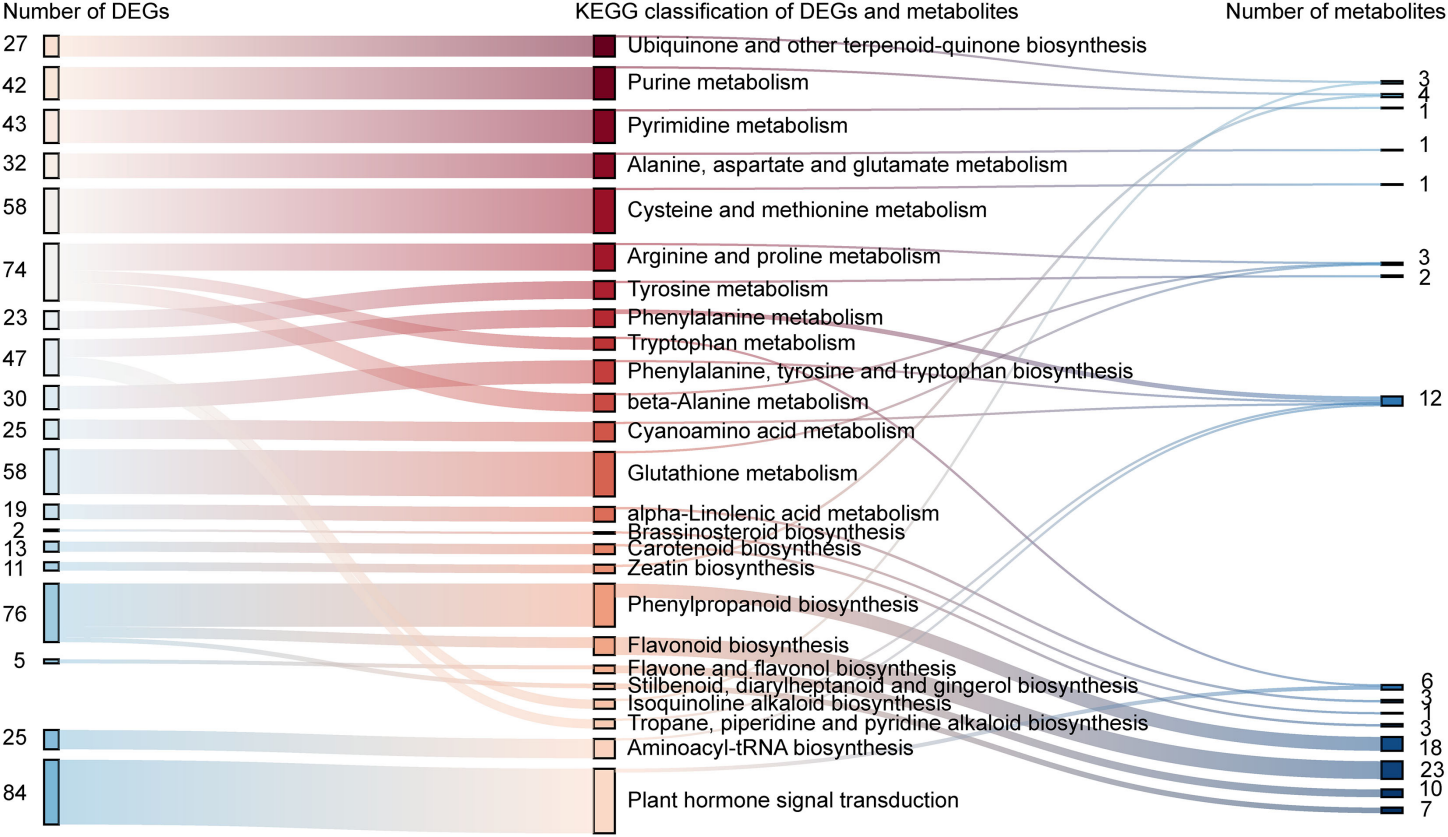
Association analysis of differential genes and differential metabolites.The data presented on the left side of the figure corresponds to the number of differentially expressed genes across various KEGG pathways, enquanto a similar data set on the right side of the figure corresponds to the number of differentially expressed metabolites.

**Figure 6:**
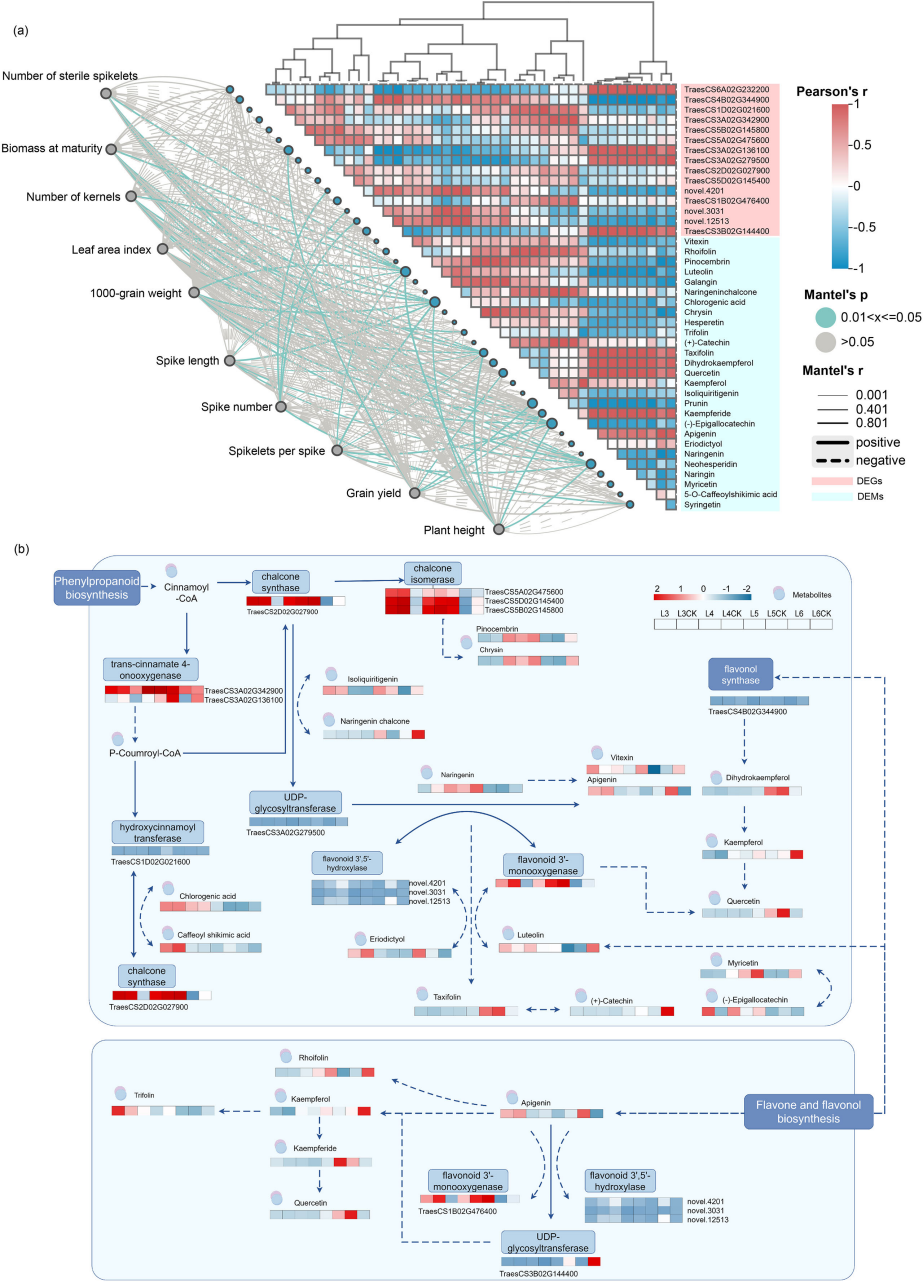
Correlation analysis between winter wheat phenotypes, differentially expressed genes (DEGs), and differentially expressed proteins (DEMs) **(a)**, and their involvement in the flavonoid biosynthesis pathway **(b)**.

Transcriptomic and metabolomic analyses revealed that delayed rehydration was associated with flavonoid metabolism (**Figure 5**). A total of 19 DEGs and 33 differentially expressed metabolites (DEMs) were associated with flavonoid and flavonol biosynthesis. Significant correlations were observed between the majority of molecular components through interactive network analysis, indicating coordinated regulation of flavonoid synthesis and metabolic processes (**Figure 6a**, **Supplementary Table S1**). Notably, the dihydrokaempferol-related gene TraesCS4B02G344900, involved in phenylpropanoid metabolism, flavonol synthase, and flavonol biosynthesis, demonstrated significant positive correlations with 12 metabolites, including naringenin, chrysin, taxifolin, and prunin. The UDP-glycosyltransferase gene TraesCS3A02G279500 exhibited strong positive correlations with both myricetin- and naringenin-related metabolites. Subsequent association analysis between agronomic traits and differential genes, as well as metabolites, demonstrated that yield exhibited a significant positive correlation with quercetin and prunin (**Figure 6a**, **Supplementary Table S1**). Notably, the number of panicles showed a positive correlation with flavonoid 3’,5’-hydroxylase (novel.12513) and syringetin. Chlorogenic acid and luteolin exhibited significant positive correlations with multiple agronomic traits, involved in biomass at maturity, number of kernels, 1000-grain weight, spike length, and plant height.

DEGs and DEMs involved in flavonoid metabolism-related pathways were conducted under both rehydration and drought conditions (**Figure 6b**, **Supplementary Table S1**). Transcript analysis revealed consistent downregulation of all chaconase- and chaconase isomerase-related DEGs in the L4 treatment, corresponding to downregulation in DEMs (including pinocembrin, chrysin, chlorogenic acid, and caffeoyl shikimic acid). Most of the flavonol synthase-related genes (TraesCS4B02G344900, TraesCS3A02G279500, and novel 3031) were downregulated or remained unchanged in L3/L5 but were upregulated in L4/L6. Luteolin, which is involved in flavonoid 3’-monooxygenase, flavanol synthase, and flavanol biosynthesis, increased in L3 and L4 with early rehydration but decreased with rehydration postponed to L5. Similarly, apigenin decreased sharply at L4, as did the gene encoding flavonoid 3’-monooxygenase (TraesCS1B02G47640).

## 4 Discussion

### 4.1 Characteristics of water consumption in winter wheat upon postponed irrigation

Soil water content is a critical factor in regulating plant growth and development, and plant drought tolerance varies by growth stage. Typically, the distribution curves of mean soil water content displayed bimodal distribution characteristics, with the initial peak and valley appearing at 10–20 and 50 cm depths, respectively, followed by secondary extrema at 100 and 280 cm soil layer (Li et al., 2017). Maximum moisture accumulation occurred predominantly within the top (20–40 cm) and middle (40–60 cm) layers, accounting for 60% of the estimated total evaporation (ETc). Existing literature indicated that the deep soil water reserves critically influence the supply for wheat development. Field measurements confirmed strong correlations with winter wheat growth and yield formation. Previous research has established the regulatory function of shallow soil moisture at 0–60 cm as a primary environmental control, with documented variations across different irrigation periods (**Figure 1a**). The maximum irrigation depth is maintained at 50 cm, thereby ensuring an optimally moist soil layer from the surface to a depth of 50 cm, which supports the normal growth of winter wheat. This strategy also encourages deeper root penetration, allowing plant roots to access water from a more profound soil profile(Wu P. N. et al., 2024). In contrast, the irrigation treatment extends the maximum irrigation depth to 80 cm. Under these conditions, winter wheat may exhibit a phenomenon known as ‘root laziness,’ where the roots are less inclined to penetrate deeper soil layers. Instead, they tend to depend on the moisture and nutrients available in the more readily accessible, shallow soil layer (Zhao et al., 2023).

From the perspective of the irrigation period, upper and lower limits of relative soil moisture content based on root-sourced signal characteristics of plant determine the amount and timing of regulated deficit irrigation (RDI). SWC measurements recorded values of 60.3% and 51.3% at the onset of root-sourced signaling at the jointing stage, and 65.2% and 56.3% at the terminal heading stage (Ma et al., 2019). Post-heading irrigation delay maintained sufficient water reserves within the 0–200 cm soil layer after flowering, potentially preventing the initiation of the water deficit signal in the roots (**Figure 1a**). Jointing-stage soil water storage enhancement ranging from 4.3% to 8.0% demonstrated optimized water utilization efficiency through preferential deep-layer extraction during critical growth stages (Xing et al., 2025). The primary objective of irrigation is to maximize crop yields for a certain amount of water consumption and greatly improve crop water productivity by reducing water consumption. Deficit irrigation strategies applied during different fertility periods demonstrated significant water conservation effects. Implementation of RDI from tillering through early spring growth until stem elongation completion resulted in a reduction in water consumption while increasing water productivity (WP) of wheat (Ma et al., 2016). Controlled moisture stress between 55% and 75% of full capacity (FC), followed by post-anthesis recovery to 75%-85% FC after anthesis, can improve the WUE by 5%-22% (Mu et al., 2021). Bao et al. (2024) reported optimal irrigation management, maintaining 75% of ETc effectively reduced both soil water consumption and evapotranspiration while significantly improving irrigation WP. Postponed rehydration prompted a decline in overall water consumption for all experimental groups throughout the reproductive phase. All treatments featuring postponed rehydration during the interval between jointing and flowering exhibited decreased water utilization (**Figure 1b**). Controlling soil moisture levels during critical periods of crop water demand helps regulate water consumption and allocation, resulting in improved water use efficiency (WUE) in winter wheat cultivation systems.

### 4.2 Adaptive regulation of agronomic traits of winter wheat to water deficit and rehydration

Long-term soil water stress permanently shrinks crop structural parameters, including plant height, leaf area expansion, canopy cover, and internode length. Controlled water stress during the recovery-jointing stage enhances canopy structure optimization and promotes pre-anthesis assimilate partitioning toward developing grains. While sustained moderate stress across all growth stages facilitates dry matter translocation, the concomitant reduction in post-anthesis biomass accumulation ultimately compromises final grain yield (Liu et al., 2016). Delayed irrigation at all four stages consistently diminished three key maturity parameters: LAI, plant height, and BAM. However, delayed rehydration to the fourth leaf stage in spring did not significantly impact the phenotypic characteristics of winter wheat (**Figure 2a, c**). Plants experiencing mild to moderate water deficits were more successful in translocating pre-anthesis assimilates. Excessive water stress significantly impaired pre-anthesis assimilate redistribution in wheat plants (Zhuang et al., 2024). Although reducing the photosynthetic area of the wheat canopy leads to adverse effects on dry matter accumulation and final yield under limited irrigation, radiation remains distributed in both the upper and lower foliage layers. Such optimized light distribution partially compensates for yield losses associated with reduced total photosynthetic capacity by improving the light use efficiency of leaves and other organs (Zhao et al., 2020).

Previous research demonstrates that regulated irrigation protocols significantly decrease three key vegetative parameters: leaf area, plant height, and the number of tillers, thereby reducing canopy-level water consumption (Zhang et al., 2018). During the initial growth phase of winter wheat (prior to the jointing stage), the L3 irrigation regime achieved maximal stem density (**Figure 2b**). Conversely, the L4 treatment maintained superior tiller preservation capacity, sustaining an elevated number of stems in the L4 treatment until maturity. Such structural modifications in the crop canopy diminish both transportational water loss and photosynthetic fluxes, while simultaneously enhancing light capture efficiency and radiation utilization within the optimized canopy structures (Perdomo et al., 2015). Crop water indicators (at both the leaf and root levels) were unresponsive to soil water variation during the later growth stages of winter wheat (after heading). The canopy essentially ceased to develop further, with soil moisture having a greater impact on grain formation and development than on crops (Zhang et al., 2018). Accordingly, limited irrigation represents an effective water conservation approach for agricultural production in moisture-limited environments, particularly in semi-arid regions.

### 4.3 The effect of soil water dynamics on Spike development and yield in wheat

The period from jointing to booting is a critical stage for wheat development and degradation, and floret development tends to be highly susceptible to drought conditions. The number of grains per spike is the manifestation of wheat floret development, degradation, and setting (Yu et al., 2019). Pre-reproductive drought stress significantly exacerbated the degradation and abortion of wheat florets. Quantitative analysis revealed 5.3%-8.0% and 8.3%-9.0% reductions in the number of fertile florets and grains per spike, respectively (Li et al., 2024). In the present study, irrigation postponed until the four-leaf stage did not considerably reduce the number of grains per spike, whereas irrigation postponed until the five-leaf stage and increasing drought stress severity caused pronounced reductions in grain number (**Figures 3a–d**). Post-floret differentiation water deficit substantially enhances the degradation of florets and pollen sterility, ultimately reducing the number of grains per spike (Dong et al., 2017). Conversely, jointing-stage irrigation with 60mm water application 10 days post-jointing significantly increases the number of spikes and the kernel numbers per spike, thereby boosting winter wheat productivity (Fan et al., 2019). Nevertheless, the intricate physiological mechanisms governing floret-to-grain transition remain incompletely characterized. Furthermore, the differential capacity of wheat plant spikes and stems to absorb assimilates in plants with variable drought tolerance levels warrants comprehensive investigation.

In general, the reduction in crop yield caused by severe water stress has been primarily attributed to a reduction in the number of tillers per spike, with subsequent reductions in the number of spikes per unit area (Zhuang et al., 2024). However, moisture conditions during critical growth phases modulate tiller dynamics in winter wheat, with jointing-stage moisture restriction suppressing nonproductive tiller development and heading-stage conditions accelerating their senescence (Wu et al., 2023). Observations from this study demonstrate that controlled water deficit during vegetative growth phases enhances the number of spikes through selective elimination of nonproductive tillers, constituting the principal determinant of final yield (**Figure 2b**). This phenomenon aligns with documented grain yield compensation mechanisms following post-stress rehydration during reproductive development (Zhang et al., 2018). Spike number in dry years was positively correlated with pre-anthesis water usage from 80 to 160 cm depth, while the kernel number was correlated with balanced pre- and post-anthesis allocation within the 0–200 cm layer in normal years. Under high precipitation conditions, post-anthesis water uptake from the 160–200 cm soil depth positively influenced 1000-grain weight, demonstrating a synergistic yield enhancement effect (Xing et al., 2025). The 1000-grain weight of winter wheat increased progressively with delayed irrigation, with this parameter constituting the principal yield-limiting factor for the L3 treatment (**Figures 3f, g**). Irrigation treatments targeting the fifth and sixth leaves in spring negatively impacted grain yield due to significant declines in spike and grain number (**Figures 3e, g**).

Strategic allocation of restricted irrigation during crucial growth stages typically achieves consistent winter wheat yields while optimizing water resource utilization (Yang et al., 2024). Current agronomic recommendations prioritize the early growth stage irrigation applications, as this practice demonstrates minimal yield penalty while substantially enhancing water production efficiency. The heightened sensitivity of cereal crops to hydric stress during vegetative development phases is quantitatively reflected in elevated cumulative crop water stress index (CCWSI). As evidenced by a reduction in CCWSI, wheat is less sensitive to a water deficit during the mid- and late growth stages (Wen et al., 2017). Water application during either the flag leaf stage or post-flowering improves canopy structure and boosts grain production (He et al., 2023; Mohammadi, 2024). Anthesis-stage supplemental irrigation demonstrates particularly pronounced yield benefits when cumulative precipitation between jointing and anthesis falls below 31.9 mm (Li et al., 2023). Notably, experimental evidence confirms the yield-enhancing effect of four-leaf-stage irrigation in winter wheat cultivation systems. The proposed mechanism involves spring irrigation targeting the fourth leaf stage, which effectively reconciles early and late drought stress. This combination of factors led to better coordinated yield components and the maximum grain yield (**Figure 3g**). We propose that spring irrigation at the 4th leaf stage effectively mitigated early and late drought stress, with pre-irrigation moderate drought stress playing a positive regulatory role. This synergy led to better yield component coordination and the highest grain yield. The results suggest that rehydration after drought stress compensates for storage and accumulation in grains, enhancing assimilate transfer and carbohydrate metabolism post-flowering, thus boosting grain filling and final yield(X. Liu et al., 2024).

### 4.4 Flavonoid metabolism-related pathways in winter wheat response to rehydration

Flavonoids function as negative regulatory agents of auxin translocation in plant systems, consequently affecting vertical growth patterns. These secondary metabolites additionally mediate photoassimilate redistribution from the stem to the ear, enhancing floret survival and increasing grain number and yield (Gao et al., 2023). Maize exhibits drought-responsive upregulation of stem-expressed genes associated with abscisic acid, lignin, flavonoid biosynthesis, and carbon metabolism in the stem, concurrently suppressing stem elongation and promoting the allocation of assimilates to the ear (Gao et al., 2023). Rice enhances tolerance to drought stress by activating the phenylpropanoid pathway, triggering transcriptional induction of multiple biosynthetic genes such as phenylalanine ammonia-lyase 4, cinnamyl alcohol dehydrogenase 6, and 4-coumarate-CoA ligase-like 6 (Shim et al., 2024). The increased biosynthetic capability of amino acids and the ability to scavenge ROS, resulting from higher antioxidant activities and increased flavonoids, may be the mechanisms underlying wheat’s drought tolerance. The rapid recovery of wheat may be due to its ability to recover from reversible ROS damage and rapidly synthesize amino acids (Lv et al., 2022). Interactive correlation analysis revealed that the majority of differential genes and metabolites associated with flavonoid synthesis and metabolism exhibited significant correlations. These observations align with the results of lower plant height and higher crop yields (**Figures 3g**; **Figure 6a**; **Supplementary Table S1**). Under abiotic stress conditions, metabolic channeling from lignification pathways toward flavonoid biosynthesis facilitates flavonoid glycoside accumulation, conferring stress protection in rice cultivars. Overexpression of flavonoid glycosides is associated with larger grains and enhanced tolerance to abiotic stress (N. Q. Dong et al., 2020; Zou et al., 2021). Our experimental results elucidate mechanistic relationships between metabolic flux modulation, grain morphology regulation, and stress tolerance, presenting viable strategies for crop improvement through metabolic engineering approaches.

Particularly, a previous study has established the critical role of phenylpropanoid biosynthesis in enhancing drought tolerance (Sun et al., 2025). After drought stress treatment, high levels of different amino acids, alkaloids, organic acids, and flavonoids in the drought-treated wheat indicate strong drought-tolerant capacity (X. Y. Guo et al., 2020). The dihydrokaempferol-related gene TraesCS4B02G344900, functioning within phenylpropanoid metabolism, flavonol biosynthesis, and flavonol synthase activity, demonstrated strong positive correlations with 12 metabolites, including naringenin, chrysin, taxifolin, and prunin (**Figure 6a**), **Supplementary Table S1**). Comparative expression profiling of flavonoid metabolism-associated DEGs and DEMs was conducted across rehydration and drought conditions (**Figure 6b**, **Supplementary Table S1**). Through integrated metabolomic and transcriptomic analyses, a significant up-regulation of metabolites and genes associated with the biosynthesis of flavonoids and phenolic acids, including HCT, FLS, CHS, and F3’5’H, was observed in wheat subjected to drought stress. The findings suggest that the metabolic pathways of flavonoids and phenolic acids are linked to the drought resistance of wheat seedlings. The differentially expressed metabolites and genes involved in their biosynthesis may serve as crucial determinants underlying variations in drought tolerance (X. R. Guo et al., 2025). All DEGs related to chalcone synthase and chalcone isomerase were significantly down-regulated in L4, while DEMs, including pinocembrin, chrysin, chlorogenic acid, and caffeoyl shikimic acid, were down-regulated. Luteolin, a metabolite participating in flavonoid 3’-monooxygenase activity, flavonol synthase activity, and flavonol production activity, exhibited elevated concentrations in L3 and L4 with early rehydration but decreased with postponed rehydration to L5. Transcriptional alterations are hypothesized to drive the substantial accumulation of phenolic compounds, such as flavonoids, in plant leaves, effectively lowering levels of reactive oxygen species. The activities of phenylalanine ammonia-lyase (PAL), cinnamate 4-hydroxylase (C4H), and 4-coumarate-coenzyme A ligase (4CL) enzymes, along with the total phenolic and flavonoid contents (TPC and TFC, respectively), were significantly increased in spike organs under drought stress. Additionally, drought conditions stimulated the expression of structural genes (TaPAL, TaC4H, Ta4CL, TaCHS, TaCHI, TaFNS, TaF3H, TaFLS, TaDFR, and TaANS) associated with the phenylpropanoid pathway in spike organs during the middle and late grain filling stages. This suggests that the enhanced drought tolerance observed in spike organs is linked to the upregulation of the phenylpropanoid pathway, which may contribute to improved water retention, relatively higher photosynthetic activity, and reduced membrane damage (X. R. Li et al., 2020).

## 5 Conclusions

Delayed rehydration implementation reduces total water consumption, particularly during the jointing-to-flowering developmental phase in winter wheat. This water management strategy led to significant reductions in plant height, LAI, and BAM in winter wheat. However, grain yield demonstrated enhancement through three yield component modifications: higher number of spikes, a more balanced number of grains, and an increased 1000-grain weight. Transcriptomic and metabolomic analyses revealed that delayed rehydration is associated with flavonoid metabolism. The collective evidence points to flavonoid metabolism potentially serving as a molecular regulator of this critical developmental stage.

## Data Availability

The datasets presented in this study can be found in online repositories. The names of the repository/repositories and accession number(s) can be found in the article/**Supplementary Material**.
