# Supplementary material for: Flavonoid metabolism is involved in regulating the growth of winter wheat upon rehydration
Source: Front Plant Sci. 2026 Feb 2;16:1693576. doi: 10.3389/fpls.2025.1693576 (PMC12907160; doi:10.3389/fpls.2025.1693576)
Supplement: Supplementary file 1 [file Table1.docx]

Supplementary Material

**Table S1.** Differentially Expressed Genes in the Flavonoid Metabolic Pathway and Detailed Information

| **ID** | **L3** | **L3_CK** | **L4** | **L4_CK** | **L5** | **L5_CK** | **L6** | **L6_CK** | **tf_family** | **pathway_id** | **pathway** |
| --- | --- | --- | --- | --- | --- | --- | --- | --- | --- | --- | --- |
| TraesCS6A02G232200 | 2.35147 | 3.461656 | 0.937291 | 0.926141 | 0.686617 | 7.230938 | 12.13559 | 2.162444 | - | osa00940\|\|osa00941\|\|osa00945\|\|osa01100\|\|osa01110 | Phenylpropanoid biosynthesis\|\|Flavonoid biosynthesis\|\|Stilbenoid, diarylheptanoid and gingerol biosynthesis\|\|Metabolic pathways\|\|Biosynthesis of secondary metabolites |
| TraesCS4B02G344900 | 0.343628 | 0.599601 | 0.457005 | 0.369276 | 0.367733 | 0 | 0 | 0.226602 | 2OG-FeII_Oxy | osa00941\|\|osa01100\|\|osa01110 | Flavonoid biosynthesis\|\|Metabolic pathways\|\|Biosynthesis of secondary metabolites |
| TraesCS1D02G021600 | 0.656704 | 1.042315 | 0.340669 | 0.619951 | 1.888622 | 0.037039 | 0 | 0.59268 | - | osa00940\|\|osa00941\|\|osa00945\|\|osa01100\|\|osa01110 | Phenylpropanoid biosynthesis\|\|Flavonoid biosynthesis\|\|Stilbenoid, diarylheptanoid and gingerol biosynthesis\|\|Metabolic pathways\|\|Biosynthesis of secondary metabolites |
| TraesCS3A02G342900 | 16.84525 | 20.02022 | 6.351833 | 29.06151 | 36.07315 | 57.15369 | 18.517 | 5.776846 | p450 | osa00130\|\|osa00940\|\|osa00941\|\|osa00945\|\|osa01100\|\|osa01110 | Ubiquinone and other terpenoid-quinone biosynthesis\|\|Phenylpropanoid biosynthesis\|\|Flavonoid biosynthesis\|\|Stilbenoid, diarylheptanoid and gingerol biosynthesis\|\|Metabolic pathways\|\|Biosynthesis of secondary metabolites |
| TraesCS5B02G145800 | 29.83644 | 50.18718 | 1.843384 | 15.01277 | 29.296 | 41.09822 | 9.133878 | 0.173065 | - | osa00941\|\|osa01100\|\|osa01110 | Flavonoid biosynthesis\|\|Metabolic pathways\|\|Biosynthesis of secondary metabolites |
| TraesCS5A02G475600 | 13.03552 | 18.27521 | 2.381189 | 5.352581 | 7.633911 | 13.30624 | 5.395466 | 1.18644 | - | osa00941\|\|osa01100\|\|osa01110 | Flavonoid biosynthesis\|\|Metabolic pathways\|\|Biosynthesis of secondary metabolites |
| TraesCS3A02G136100 | 3.806105 | 7.515749 | 0.660497 | 3.735226 | 6.933951 | 46.73478 | 19.82339 | 0.618805 | p450 | osa00130\|\|osa00940\|\|osa00941\|\|osa00945\|\|osa01100\|\|osa01110 | Ubiquinone and other terpenoid-quinone biosynthesis\|\|Phenylpropanoid biosynthesis\|\|Flavonoid biosynthesis\|\|Stilbenoid, diarylheptanoid and gingerol biosynthesis\|\|Metabolic pathways\|\|Biosynthesis of secondary metabolites |
| TraesCS3A02G279500 | 0.504899 | 0.371801 | 0.09574 | 0.414117 | 0.237492 | 1.148606 | 0.996069 | 0.345555 | UDPGT | osa00941\|\|osa01110 | Flavonoid biosynthesis\|\|Biosynthesis of secondary metabolites |
| TraesCS2D02G027900 | 28.74107 | 34.6272 | 1.704252 | 12.74038 | 26.3543 | 66.5669 | 8.539764 | 0.016028 | Chal_sti_synt_N | osa00941\|\|osa01100\|\|osa01110\|\|osa04712 | Flavonoid biosynthesis\|\|Metabolic pathways\|\|Biosynthesis of secondary metabolites\|\|Circadian rhythm - plant |
| TraesCS5D02G145400 | 26.05045 | 46.71003 | 1.406904 | 10.24175 | 18.88667 | 47.1193 | 9.62954 | 0.359277 | - | osa00941\|\|osa01100\|\|osa01110 | Flavonoid biosynthesis\|\|Metabolic pathways\|\|Biosynthesis of secondary metabolites |
| novel.4201 | 1.167789 | 3.114483 | 2.618255 | 1.482447 | 0.944054 | 0.031846 | 0.069239 | 2.079129 | - | osa00941\|\|osa00944 | Flavonoid biosynthesis\|\|Flavone and flavonol biosynthesis |
| TraesCS1B02G476400 | 9.58735 | 19.95988 | 0.58619 | 5.777173 | 13.6127 | 50.33097 | 6.34547 | 0.162106 | p450 | osa00941\|\|osa00944\|\|osa01100\|\|osa01110 | Flavonoid biosynthesis\|\|Flavone and flavonol biosynthesis\|\|Metabolic pathways\|\|Biosynthesis of secondary metabolites |
| novel.3031 | 0.299653 | 1.008754 | 1.034696 | 0.403333 | 0.101472 | 0.009819 | 0.004829 | 0.435112 | - | osa00941\|\|osa00944 | Flavonoid biosynthesis\|\|Flavone and flavonol biosynthesis |
| novel.12513 | 0.884051 | 3.714457 | 1.641817 | 0.859313 | 0.667501 | 0.049811 | 0.034071 | 2.598939 | - | osa00941\|\|osa00944 | Flavonoid biosynthesis\|\|Flavone and flavonol biosynthesis |
| TraesCS3B02G144400 | 0.329631 | 0.165545 | 0.083207 | 0.099286 | 0.149799 | 10.71064 | 37.81261 | 0.3278 | UDPGT | osa00944 | Flavone and flavonol biosynthesis |
| novel.4201 | 1.167789 | 3.114483 | 2.618255 | 1.482447 | 0.944054 | 0.031846 | 0.069239 | 2.079129 | - | osa00941\|\|osa00944 | Flavonoid biosynthesis\|\|Flavone and flavonol biosynthesis |
| TraesCS1B02G476400 | 9.58735 | 19.95988 | 0.58619 | 5.777173 | 13.6127 | 50.33097 | 6.34547 | 0.162106 | p450 | osa00941\|\|osa00944\|\|osa01100\|\|osa01110 | Flavonoid biosynthesis\|\|Flavone and flavonol biosynthesis\|\|Metabolic pathways\|\|Biosynthesis of secondary metabolites |
| novel.3031 | 0.299653 | 1.008754 | 1.034696 | 0.403333 | 0.101472 | 0.009819 | 0.004829 | 0.435112 | - | osa00941\|\|osa00944 | Flavonoid biosynthesis\|\|Flavone and flavonol biosynthesis |
| novel.12513 | 0.884051 | 3.714457 | 1.641817 | 0.859313 | 0.667501 | 0.049811 | 0.034071 | 2.598939 | - | osa00941\|\|osa00944 | Flavonoid biosynthesis\|\|Flavone and flavonol biosynthesis |
